# Proximity of the neurovascular structures during all-inside lateral meniscal repair in children: a cadaveric study

**DOI:** 10.1186/s40634-018-0166-0

**Published:** 2018-12-18

**Authors:** Yi-Meng Yen, Peter D. Fabricant, Connor G. Richmond, Aleksei B. Dingel, Matthew D. Milewski, Henry B. Ellis, Philip L. Wilson, Stephanie W. Mayer, Theodore J. Ganley, Kevin G. Shea

**Affiliations:** 1 Boston Children’s Hospital, Division of Sports Medicine, Department of Orthopaedics, Harvard Medical School, Boston, MA USA; 2 Hospital for Special Surgery, New York, NY USA; 3 Texas Scottish Rite Hospital, University of Texas Southwestern, Dallas, TX USA; 4 Children’s Hospital Colroado, Aurora, CO USA; 5 Children’s Hospital Philadelphia, University of Pennsylvania School of Medicine, Philadelphia, PA USA; 6 Department of Orthopedic Surgery, Stanford University, Stanford, CA USA; 7 University of New England, College of Osteopathic Medicine, Biddeford, ME USA

## Abstract

**Purpose:**

Meniscal repair has become increasingly common in a pediatric and adolescent population. All-inside repair techniques are utilized more often given their ease of insertion and decreased operative time required. However, there are possible risks including damage to adjacent neurovascular structures. The purpose of this study to was examine the proximity of the neurovascular structures during lateral meniscus repairs in pediatric specimens simulating a worst-case scenario.

**Methods:**

Ten pediatric cadaveric knees (age 4–11) were utilized and simulated lateral meniscal repair through the posterior horn of the lateral meniscus and both medial and lateral to the popliteal hiatus through the body of the lateral meniscus was performed with an all-inside meniscal repair device. The distance to the popliteal artery or peroneal nerve was measured.

**Results:**

During posterior horn repair, the average distance from the all-inside device to the popliteal artery was 1.9 mm ± 1.1 mm. There was penetration of the artery in one specimen. During repair on the medial side of popliteal hiatus, the average distance from the all-inside device to the peroneal nerve was 3.2 mm ± 2.0 mm. During repair on the lateral side of popliteal hiatus, the average distance from the all-inside device to the peroneal nerve was 12.4 mm ± 3.7 mm.

**Conclusions:**

This study demonstrates that the proximity of the neurovascular structures to the lateral meniscus in children is extremely close and at high risk during meniscal repair with all-inside devices. This study gives important data for the proximity of these structures during these repair techniques.

**Level of evidence:**

Level 5 Cadaveric Study.

## Introduction

In the pediatric and adolescent population, meniscal injuries are rising in incidence, in part due to increased athletic participation, expanded use of magnetic resonance imaging and improved recognition and diagnosis (Bellisari et al., 2011; Brown & Davis, 2006; Francavilla et al., 2014). The menisci play a critical role in shock absorption, reduction of femoro-tibial contact forces and as secondary stabilizers within the knee joint. Partial or total menisectomies can lead to early joint degeneration and resultant osteoarthritis (Lanzer & Komenda, 1990; McDermott & Amis, 2006; Shoemaker & Markolf, 1986). The trend has therefore been towards repair of meniscal tears, particularly in the pediatric and adolescent athlete.

The inside-out meniscal repair remains the gold standard for meniscal fixation, but can be associated with an increased surgical time and the need for an additional posterolateral or posteromedial incision (Lembach & Johnson, 2014; Woodmass et al., 2017). All-inside fixation has been advanced in the arthroscopic field due to the ease of implantation and decreased surgical time. The newest generation of these devices is designed to incorporate strength of the inside-out repair with the advantages of the all-inside technique (Fillingham et al., 2017). However, there is still risk of neurovascular injury during the surgical management of lateral meniscal tears. While there have been some anatomic studies on this in the adult literature (Abouheif et al., 2011; Cohen et al., 2007; Cuellar et al., 2015; Deutsch et al., 1999), to our knowledge, there are no comparable studies in younger patients. The purpose of this study was to evaluate the proximity of the neurovascular structures when using an all-inside meniscal repair device in pediatric cadaveric specimens. This was performed in a way to give the worst-case scenario during a simulated repair. Our hypothesis was that in children, the neurovascular structures are extremely close to the lateral meniscus.

## Methods

The study was performed on ten fresh-frozen cadaveric specimens that included the full knee joint from the distal half of the femur to the proximal half of the tibia including all surrounding tissues. There were 6 male cadavers and 4 female cadavers ranging from four to eleven years in age. The knees were fresh frozen and thawed which allowed for free motion at the joint.

### Specimen preparation

**Fig. 1:**
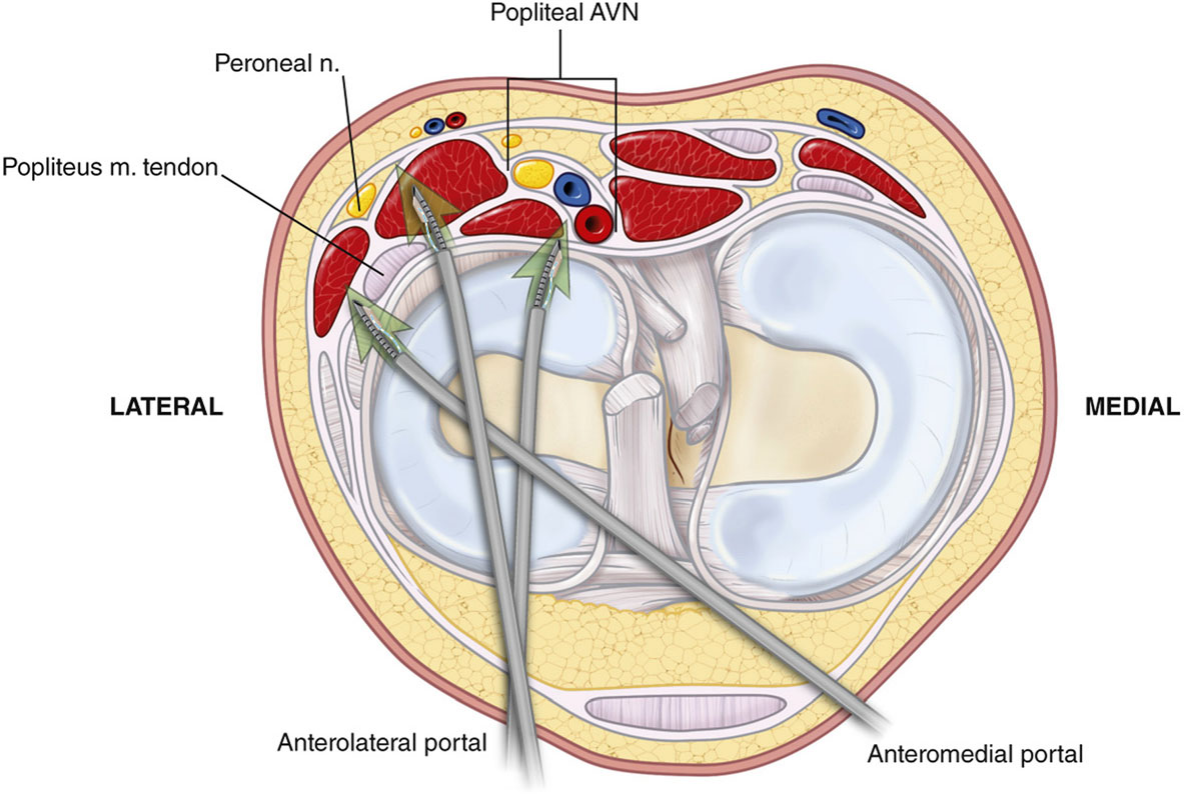
Axial schematic of the all-inside meniscal repair devices and the neurovascular structures of a left knee

All cadaveric specimens were dissected in a similar manner. The tibial plateau width and lngeth were also recorded. The knee extensor mechanism was turned downwards by freeing the quadriceps tendon and quadriceps along with and arthrotomies performed on the medial and lateral sides of the patellar tendon. The MCL and LCL were partially released to allow full visualization of the lateral meniscus. The all-inside device used for this study was a curved FasT-Fix 360° (Smith-Nephew, Andover, MA) without a depth limiter to allow for full penetration of the device (Fig. 1).

### Posterior horn of the lateral meniscus

The all inside device was placed into the joint through a simulated anterolateral portal and inserted at the visible edge (within 1-2 mm) of the posterior root of the lateral meniscus. In order to simulate a lateral meniscus tear near the posterior root, the curved tip was aimed medially and, thus, towards the neurovascular bundle. Following implantation, the tip of the needle was left in situ and then was identified through a direct posterior approach with the knee in flexion. The nearest measurement of the needle to the popliteal artery was taken.

*Medial side of popliteal hiatus.* The all-inside device was again placed into the joint through a simulated anterolateral portal and inserted at the most medial corner of the popliteal hiatus. The curved tip was aimed laterally towards the peroneal nerve. A posterolateral dissection was performed to identify the needle and tip. The nearest measurement of the needle to the common peroneal nerve was recorded.

### Lateral side of popliteal hiatus

The all inside device was placed into the joint through a simulated anteromedial arthroscopy portal and inserted at the lateral corner of the popliteal hiatus. The curved tip of the needle was aimed medially towards the common peroneal nerve. Measurement of the needle to the peroneal nerve was taken.

### Measurements

All the measurements were done by a single fellowship-trained surgeon. A digital caliper with a nominal precision of 0.01 mm was used in all measurements from the position of the needle to the neurovascular structures. Three caliper measurements were performed on each specimen at each of the three locations of the all inside device needle and the average was used as the final measure. The knee was held at 90 degrees of flexion in all cases to simulate arthroscopic positioning in the figure-of-four position.

## Results

The dimensions of the each specimen and measurements between the neurovascular structures and the all inside meniscal devices are listed in Table 1.

**Table 1:** Age of specimens, Tibial plateau dimensions, and distance from meniscal repair device to neurovascular structures. (All measurements presented in mm)

Age/Sex	Tibial Plateau Length	Tibial Plateau Width	Distance to Popliteal Artery	Distance to Peroneal Nerve (Medial Popliteus)	Distance to Peroneal Nerve (Lateral Popliteus)
4 M	30.8	20.2	0.0	1.0	5.5
4 F	30.7	20.4	1.8	0.4	15.1
7 F	30.4	20.3	1.7	4.4	8.2
9 M	60.2	30.6	2.9	5.0	17.6
9 F	50.1	30.2	1.3	3.7	11.6
9 F	50.1	20.9	2.9	1.2	13.1
9 M	60.1	30.4	0.9	1.9	10.6
10 M	Unable to collect	Unable to collect	3.8	4.4	16.3
10 M	60.4	30.8	1.4	6.6	13.4
11 M	60.7	30.9	2.1	3.0	12.7

### Distance from posterior root to popliteal artery

**Fig. 2:**
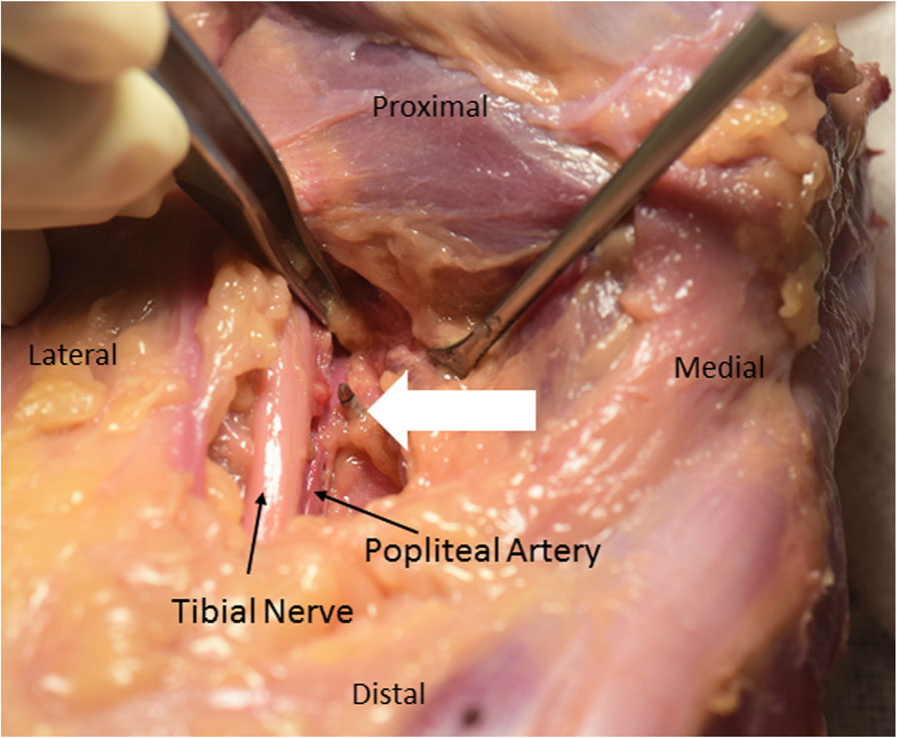
Viewing from posterior to anterior of a left knee, showing the needle tip (white arrow) just adjacent to the popliteal artery and the proximity to the tibial nerve

The average distance from the all-inside device to the popliteal artery was 1.9 mm ± 1.1 mm (SD). There was penetration of the artery in a specimen of a 4 year-old knee specimen (Fig. 2). The farthest distance to the artery was 3.8 mm in a 10 year-old knee.

### Medial side of popliteal Hiatus to the common peroneal nerve

**Fig. 3:**
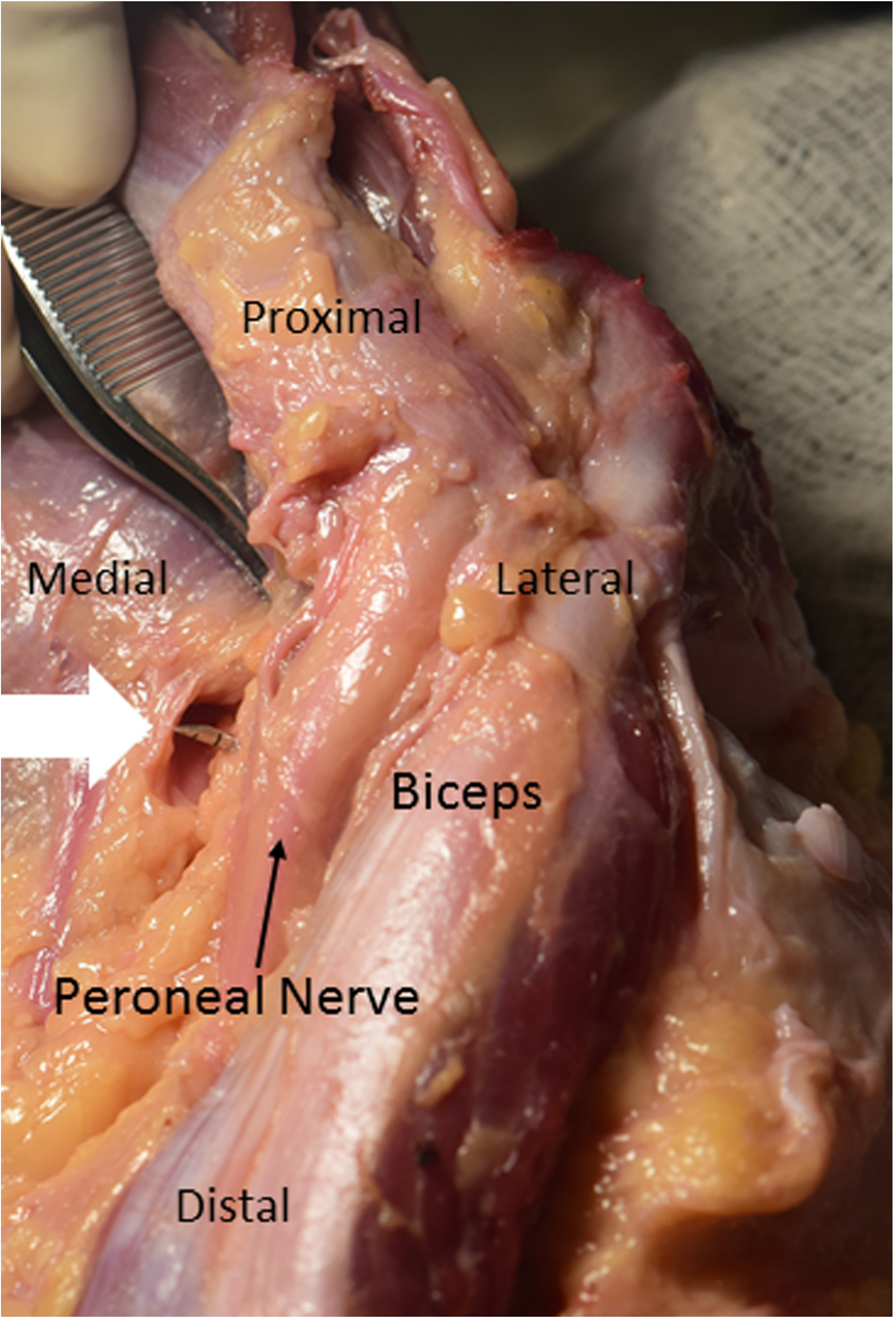
Viewing from posterior to anterior of a right knee, showing the needle tip (white arrow) adjacent to the peroneal nerve while performing a repair on the medial side of the popliteal hiatus

The average distance from the all-inside device to the peroneal nerve was 3.2 mm ± 2.0 mm (SD) (Fig. 3). The closest distance to the nerve was 0.4 mm in a 4 year-old knee specimen while the farthest distance to the nerve was 6.6 mm in a 10 year-old knee.

### Lateral side of popliteal Hiatus to the common peroneal nerve

The average distance from the all-inside device to the peroneal nerve was 12.4 mm ± 3.7 mm (SD). The closest distance to the nerve was 5.5 mm in a 4 year-old knee. The farthest distance to the nerve was 17.6 mm in a 9 year-old knee.

## Discussion

The incidence of neurovascular injury during all-inside meniscal repair is reportedly very low (Bernard et al., 1994). In a study of 375,000 knee arthroscopies, there were 9 injuries to the popliteal artery (Complications in arthroscopy, 1986). In a similar study of 118,590 arthroscopic procedures, there were 6 cases of injury to the popliteal artery. (Complications of arthroscopy and arthroscopic surgery, 1985) There have been some case reports of common peroneal nerve injury after lateral meniscal repair (Anderson & LaPrade, 2009; Krivic et al., 2003). However, to the author’s knowledge, the incidence of neurovascular complications following meniscal repair in children has not been reported. While, the overall number of procedures in children per year is lower than in adults, meniscal repair in children may be more common. As the overall dimensions of a pediatric knee are smaller, the proximity of the neurovascular structures may place them at greater risk during meniscal repair.

This study was conducted in order to determine the proximity of the neurovascular structure in a “worst-case” scenario utilizing an all-inside meniscal repair device intentionally aimed towards the neurovascular structures. If the all-inside device were utilized in as recommended, this would bring the device further from the neurovascular structures and therefore safer. The popliteal artery is located near the posterior horn of the lateral meniscus and in extension is only separated by the posterior capsule by a small layer of fat. The common peroneal nerve is located posterior to the biceps femoris tendon and is located next to the popliteal hiatus on the medial side. In adult studies, when the distance between the repair device and the neurovascular structures was 10 mm, this was considered at risk, when the distance was less than 5 mm, this risk was considered high (Abouheif et al., 2011; Cuellar et al., 2015; Deutsch et al., 1999). With this definition, lateral meniscal repair of the posterior horn near the root in children should be considered especially high risk as the popliteal artery is within 5 mm of the suturing device. Direct penetration of the artery was seen in one specimen of a 4 year-old child. The common peroneal nerve is also at high risk when repairing the meniscus at the medial edge of the popliteal hiatus. The device was essentially adjacent to the peroneal nerve in a specimen of a 4 year-old child.

There have been several studies performed in adult cadaveric specimens that examined the safety of lateral meniscal repair. Cohen et al. examined the use of 2 repair devices in the posterior horn of the lateral meniscus and found that the tip of an all-inside device without the penetration limiter was within 3 mm of the artery (Cohen et al., 2007). Abouheif et al. found damage to the popliteal vessels and peroneal nerve in 33% and 10% of their 31 knees (Abouheif et al., 2011). Cuellar et al. reported that the risk of lateral meniscal repair is safer at 90° and worse with extension of the knee (Cuellar et al., 2015). Recently, an MRI based study demonstrated the significant risk to the neurovascular structures with repair of the lateral meniscus (Gupta et al., 2018).

This study has several limitations inherent to cadaveric studies. While the insertion of the device was done at 90° to simulate the figure-of-four position during arthroscopic surgery, this was performed in a dry environment without fluid to distend the joint. The study was performed with a curved all-inside device with an angle of 29° intentionally aimed towards the neurovascular bundle. We wished to perform a “worst-case” scenario, however, if the direction of the curve of the device was altered or if a straight device was utilized, this would likely decrease the risk to the neurovascular structures. Additionally, we simulated the anterolateral and anteromedial portals by positioning the devices adjacent to the patellar tendon. Variation in true placement of these portals can alter the trajectory of the device. This study utilized the suture device without the use of the limiter which does not take into account the anterior-posterior position of the neurovascular structures. The proximity of the neurovascular structures in the antero-posterior direction is age-dependent and is as close as 4 mm (submitted) It is likely that positioning the penetration limiter less than 20 mm may decrease the risk of penetrating the peroneal nerve or popliteal artery, but this was not addressed in this study. The dissection posteriorly and posteolaterally were performed after device insertion and may have allowed for movement of the tissues. Although, the fresh freezing and thaw method allows for near normal elasticity of the tissue, dissection of the tissue may inevitably alter the true relationship of the meniscus with the neurovascular structures.

This study demonstrates that the proximity of the neurovascular structures to all-inside devices inserted in the lateral meniscus in children are closer than has been previously demonstrated in some adult studies. As the incidence of meniscal repair is rising in children, great care must be taken when performing lateral meniscal repair using all-inside devices. Future research should simulate actual arthroscopic conditions and the proper clinical use of all-inside meniscal repair devices in pediatric specimens.
